# Big five personality traits prediction with AI

**DOI:** 10.1192/j.eurpsy.2021.1189

**Published:** 2021-08-13

**Authors:** A. Mereu

**Affiliations:** Research performed independently, Cagliari, Italy

**Keywords:** traits, psychometry, Artificial Intelligence, Personality

## Abstract

**Introduction:**

Openness, conscientiousness, extroversion, agreeableness and neuroticism are known as the Big Five personality traits (BFPT). They are theoretical building blocks of the personality and comprise wide and interconnected spectra. Artificial intelligence (AI) could help to grasp their complexity.

**Objectives:**

To investigate whether AI could predict the BFPT from themselves.

**Methods:**

Data from 2,697 questionnaires were analysed using an AI. The short form of the International Personality Item Pool was used to assess the BFPT. Four of the BFPT scores were employed to predict the fifth one and the procedure was repeated for all of them alternatively. The AI was conservatively tuned to maximize the one-way random intraclass correlation coefficient (ICC) between predicted and real values. Their Pearson’s r was calculated too. The free and open source programming language R was used for all the analyses. Dataset source: Hansson, Isabelle; Berg, Anne Ingeborg; Thorvaldsson, Valgeir (2018), “Can personality predict longitudinal study attrition? Evidence from a population-based sample of older adults”, Mendeley Data, V1, doi: 10.17632/g3jx8zt2t9.1

**Results:**

Openness, conscientiousness, extroversion, agreeableness and neuroticism predictions obtained ICC of 0.219, 0.146, 0.306, 0.354, 0.121 and Pearson’s r of 0.254, 0.149, 0.393, 0.446, 0.122 respectively. The results for extroversion and agreeableness were indicative of fair performance.

**Conclusions:**

AI might be useful to predict personality traits, mainly extroversion and agreeableness. This could be utile in many situations, such as dealing with missing data or deciding whether to formally test someone. Finally, the AI used in this study is freely available, allowing anyone to experiment.

